# Variant analysis of SARS-CoV-2 strains with phylogenetic analysis and the Coronavirus Antiviral and Resistance Database

**DOI:** 10.2217/cer-2021-0208

**Published:** 2022-01-11

**Authors:** Murat Sayan, Ayse Arikan, Murat Isbilen

**Affiliations:** ^1^Kocaeli University, Research & Education Hospital, PCR Unit, 41380, Kocaeli, Turkey; ^2^Near East University, DESAM Research Institute, 99138, Nicosia, Northern Cyprus; ^3^Near East University, Department of Medical Microbiology & Clinical Microbiology, 99138, Nicosia, Northern Cyprus; ^4^Acibadem Mehmet Ali Aydinlar University, Graduate School of Health Sciences, Department of Biostatistics & Bioinformatics, 34752, Istanbul, Turkey

**Keywords:** bioinformatics, COVID-19, next-generation sequencing, phylogenetic analyses, SARS-CoV-2 variants

## Abstract

**Aims:** This study determined SARS-CoV-2 variations by phylogenetic and virtual phenotyping analyses. **Materials & methods:** Strains isolated from 143 COVID-19 cases in Turkey in April 2021 were assessed. Illumina NexteraXT library preparation kits were processed for next-generation ]sequencing. Phylogenetic (neighbor-joining method) and virtual phenotyping analyses (Coronavirus Antiviral and Resistance Database [CoV-RDB] by Stanford University) were used for variant analysis. **Results:** B.1.1.7–1/2 (n = 103, 72%), B.1.351 (n = 5, 3%) and B.1.525 (n = 1, 1%) were identified among 109 SARS-CoV-2 variations by phylogenetic analysis and B.1.1.7 (n = 95, 66%), B.1.351 (n = 5, 4%), B.1.617 (n = 4, 3%), B.1.525 (n = 2, 1.4%), B.1.526-1 (n = 1, 0.6%) and missense mutations (n = 15, 10%) were reported by CoV-RDB. The two methods were 85% compatible and B.1.1.7 (alpha) was the most frequent SARS-CoV-2 variation in Turkey in April 2021. **Conclusion:** The Stanford CoV-RDB analysis method appears useful for SARS-CoV-2 lineage surveillance.

Since its first emergence in December 2019, severe acute respiratory syndrome coronavirus (SARS-CoV-2), the causative agent of the new type of coronavirus disease 2019 (COVID-19), has had many genetic variations due to its higher mutation rates during replication. Most of these changes are not detrimental and therefore do not contribute to viral evolution [1]. These low effect or no effect changes, which are called silent amino acid changes, do not alter the basic structure and characteristics of the virus, while changes in the structural and nonstructural proteins of SARS-CoV-2 affect the viral antigenic phenotype and confer a fitness advantage. Consequently, emerging variants of SARS-CoV-2 may increase the rate of virus transmission, leading to hospitalizations and increased mortality rates in all age groups [1]. Therefore, for precise management of the ongoing COVID-19 pandemic, SARS-CoV-2 variations should be monitored.

The WHO classifies SARS-CoV-2 variants according to their genetic characteristics associated with transmissibility, increased virulence and ability to escape current diagnostic methods, vaccines and therapeutics. as variants reduce the neutralizing activity of certain monoclonal antibodies and polyclonal antibodies found in the sera of people recovering from infection [2,3]. While there are four different variants defined as alpha (501Y.V1/ B.1.1.7), beta (501Y.V2/ B.1.351), gamma (501Y.V3/P.1) and delta (lineage B.1.617) in the variant of concern (VOC) category, eta, iota, kappa and lambda have been designated as SARS-CoV-2 variant of interest (VOI) variants [2]. Detrimental variants of SARS-CoV-2 are largely caused by mutations in the *spike* glycoprotein, which mediates cell attachment and is the main target of neutralizing antibodies [3,4]. These variants continue to spread globally posing a major public health threat worldwide. As of August 17th, 2021, cases of alpha, beta, gamma and delta have been reported in 190 countries, 138 countries, 82 countries and 148 countries, respectively [5].

The more opportunity a virus has to spread, the more it will evolve. Therefore, early detection of new cases and monitoring the SARS-CoV-2 genomic sequencing for variations is significant to predict the dominant virus circulating within the population, monitor how SARS-CoV-2 changes over time into new variations that might impact health and update the geographic distribution of variants [6,7]. While SARS-CoV-2 can be detected either by detection of viral nucleic acid, mainly by reverse transcriptase real-time polymerase chain reaction assay (RT-qPCR), or detection of the presence of viral antigen or antibodies against these antigens [8], these tests cannot discriminate variants. Currently, PCR-based variant screening diagnostic assays are widely used in routine diagnostic settings for tracking these variants; however, gene analysis of whole or partial *spike* sequencing is the most accurate approach to identify variants associated with a specific trait or population [9]. Comprehensive analysis by next-generation sequencing (NGS) and bioinformatics for the ongoing genomic surveillance of SARS-CoV-2 enables the monitoring of viral spread, evolution and variation patterns worldwide in the fight against COVID-19 [10–12].

Phylogenetic analysis is widely viewed as the gold standard in genomic epidemiology [13–15]. However, with the rapid design of new virtual phenotyping technologies, identification of SARS-CoV-2 mutations can be achieved in a short time and at a low cost. Of these, the Coronavirus Antiviral and Resistance Database (CoV-RDB) by Stanford University that is freely accessible [16], has been designed to promote the comparisons between different candidate compounds against COVID-19, as well as rapid large-scale identification of SARS-CoV-2 mutations, since August 2020 [17]. CoV-RDB explores nucleotide sequences utilizing predetermined consensus SARS-CoV-2 sequences. When performing analysis with CoV-RDB, according to instructions from the database, it is recommended to input the sequences as plain text if only one sequence is analyzed and use the FASTA format if more than one sequence is submitted. The upper limit is currently given as 100 sequences containing ∼30,000 nucleotides per sequence by CoV-RDB. Although CoV-RDB is currently available for clinical diagnosis, its variant diagnostic performance has not been well assessed. The objectives of this study were to reveal the genomic characterization of SARS-CoV-2 by NGS in Turkish patients infected with COVID-19 and identify nucleotide variations by phylogenetic analysis and CoV-RDB virtual phenotyping.

## Materials & methods

### Ethical approval

The ethical approval of this study was received from the Near East University Scientific Research Ethics Committee (decision number: 1383 NEU/2021/93).

### Sample selection

In total, 143 SARS-CoV-2 strains isolated from SARS-CoV-2 infected cases in Kocaeli, Istanbul and Ankara in Turkey, at the beginning of April 2021, were included in the study. These strains were included in the study because they were screened with PCR variant screening kits and distinguished as probable SARS-CoV-2 variants.

### SARS-CoV-2 real-time polymerase chain reaction

A fully automatic rotary nucleic acid magnetic particle extraction system, the Auto Extractor GeneRotex96 (Tianlong Science and Technology Co. Xi'an City, China) was used for SARS-CoV-2 RNA isolation from the nasal/oropharyngeal swab samples. In SARS-CoV-2 diagnosis, a routine RT-qPCR kit that targets double gene (BioSpeedy, Bioeksen Inc, Istanbul, Turkey) was used that is officially preferred by the Ministry of Health in pandemic conditions.

### SARS-CoV-2 variant screening polymerase chain reaction

Two variant-specific screening PCR kits (BioSpeedy SARS-CoV-2 N501Y/variant plus kit, Bioeksen Inc., İstanbul, Turkey and Diagnovital SARS-CoV-2 N501Y, delHV 69-70, E484K mutation detection kit, RTA Laboratories Inc., Istanbul, Turkey) were used in this study. Consensus positive strains on the variant PCR screening kits were chosen for NGS.

### SARS-CoV-2* spike* next-generation sequencing polymerase chain reaction

SARS-CoV-2 real-time PCR products were purified using a NucleoFast 96 PCR kit (Macherey-Nagel GmbH, Dueren, Germany) and quantitated in spectrophotometry (Nanodrop N1000, Thermo Fisher Inc., MA, USA). The nucleic acid concentration was 0.2 ng/ul in the sample. Standardized samples were processed by NexteraXT (Illumina Inc, CA, USA) for NGS. According to the SARS-CoV-2 Wuhan Hu-1 isolate (MN908947.3 GenBank accession number), the *spike* glycoprotein receptor binding domain between 21709–23193 bps was targeted. Between 118F–1652R primers zone (∼1500 bp) was sequenced. The sequence primer pairs were R: 5′-acacctgtgcctgttaaacca-3′ and F: 5′-gacaaagttttcagatcctcagttttaca-3′ [18]. NGS was carried out on the Miseq (Illumina Inc, CA, USA) platform. The *spike* NGS PCR amplification protocol was executed in the following conditions: 45°C for 10 min, 95°C for 2 min, then for 40 cycles; 95°C for 10 s, 57°C for 30 s, and 72°C for 30 s.

Alignment of the resulting sequences was performed with Miseq Reporter based on BWA software [19]. The analysis of the sequenced data was fitted to the reference genome with BWA software, then analyzed with BaseRecalibrator and ApplyBQSR programs recommended by the Genome Analysis Tool Kit (GATK; Broad Institute, Inc. MA, USA; open source under a BSD 3-clause “New or Revised” license) and refitted according to base-read quality. Variant calling was performed with the Haplotype Caller program and variants with mapping quality below 50, a reading depth below 15 and a variant quality (QUAL) below 500 were eliminated from the analysis with the Variant Filtration program. The sequences of the samples for this region were created by modifying the mutations detected in the reference genome.

### Phylogenetic analysis

The neighbor-joining Kimura 80 distance method was performed with other sequences from all SARS-CoV-2 variants from the GeneBank database by using CLC sequence viewer 8.0 software (Qiagen, CLC bio A/S, Aarhus, Denmark). Bootstrap support values were chosen from 1000 replicates in phylogenetic tree construction. Because of numerous samples, the phylogenetic tree has been constructed as circular and rooted. The consensus reference sequence of SARS-CoV-2, MN908947.3, SARS-CoV-2 Wuhan-Hu-1, was used in this study and is available from the GenBank database [20].

### Virtual phenotyping

CoV-RDB/SARS-CoV-2 Mutations Analysis by Stanford University [21] was used to explore the nucleotide sequences of the SARS-CoV-2 strains with the consensus SARS-CoV-2 reference sequence and identify SARS-CoV-2 mutations of the *spike* gene. The obtained SARS-CoV-2 variants/lineages were designated according to the WHO categorization and Centers for Disease Control and Prevention (CDC) SARS-CoV-2 Variant Classification and Definitions [22].

## Results

One hundred and forty-three *spike* gene sequences were included in the study. The sequenced data were analyzed for variations using phylogenetic analysis and virtual phenotyping. Phylogenetic analysis can reveal detailed genomic characterization and evolutionary development of organisms. As the most accurate gene tree rooting method, the SARS-CoV-2 variations obtained using the newly designed CoV-RDB were compared with phylogenetic analysis. Based on the variant classification, 109 (76%) and 122 (85%) SARS-CoV-2 variations were reported by phylogenetic analysis and CoV-RDB, respectively. Of these variations detected by CoV-RDB, n = 15, 10% were missense mutations.

**Figure 1.**
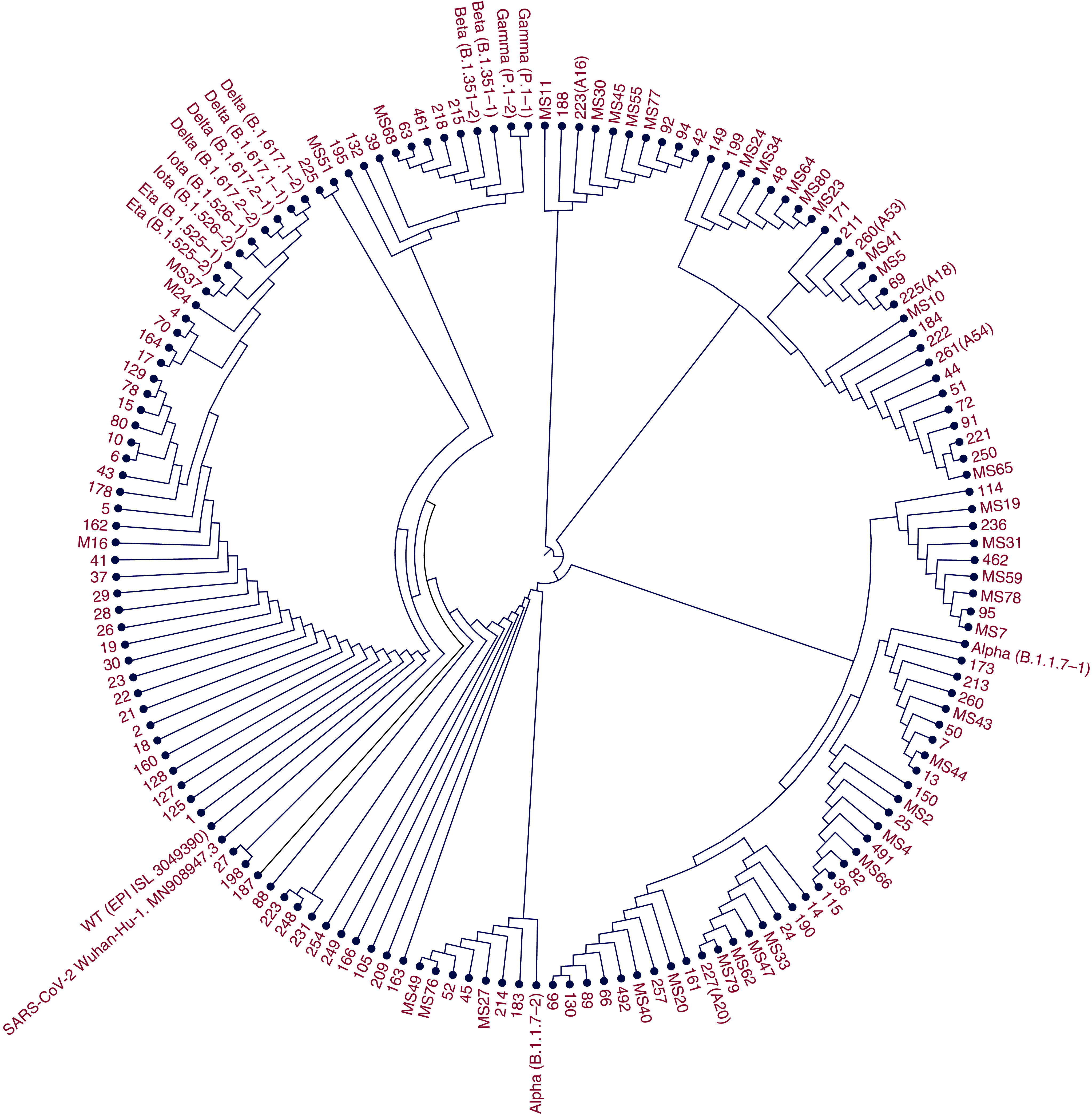
Phylogenetic tree of SARS-CoV-2 *spike* gene (1485 bp) region. The neighbor-joining tree construction method and Jukes-Cantor nucleotide distance measures were carried out with other sequences from all reference lineages from GISAID using CLC Sequence Viewer 8.0 (Qiagen, Aarhus A/S, Denmark) software. Bootstrap values are arranged as 1000 replicates. Because of numerous samples, the phylogenetic tree has been constructed as circular and rooted. The GISAID accession numbers of SARS-CoV-2 lineages are: alpha B.1.1.7-1 (EPI_ISL_3098706), alpha B.1.1.7-2 (EPI_ISL_3098709), delta B.1.617.1-1 (EPI_ISL_ 3066844), delta B.1.617.1-2 (EPI_ISL_ 3066436), delta B.1.617.2-1 (EPI_ISL_3098714), delta B.1.617.2-2 (EPI_ISL_3098716), beta B.1.351-1 (EPI_ISL_3098444), beta B.1.351-2 (EPI_ISL_3098450), eta B.1.525-1 (EPI_ISL_3089259), eta B.1.525-2 (EPI_ISL_3089260), gamma P.1-1 (EPI_ISL_3091694), gamma P.1-2 (EPI_ISL_3092082), lota B.1.526-1 (EPI_ISL_3092079), lota B.1.526-2 (EPI_ISL_3098714., WT: Wild type (EPI_ISL_3049390). The GenBank accession number of SARS-CoV-2 Wuhan-Hu-1 isolate: MN908947.3.

While the variations were obtained as lineages by phylogenetic analysis, CoV-RDB provided the mutation patterns and protein substitutions in addition to the lineages. Figure 1 illustrates different lineages obtained by the neighbor-joining method and Table 1 provides data on lineages identified by phylogenetic analysis and CoV-RDB. Mutation patterns and amino acid substitutions were also identified by CoV-RDB in SARS-CoV-2 variations.

**Table 1.** The distribution of distinct and missense mutations using phylogenetic analysis and the Coronavirus Antiviral and Resistance Database.

Phylogenetic analysis		CoV-RDB analysis		
Lineage		Mutation pattern/protein substitution	WHO lineage	WHO label
Similarity (n = 121, 85%)				
Patient	Alpha (B.1.1.7)			
MS79	B.1.1.7-2	Δ69–70, Δ144, N501Y	B.1.1.7	Alpha
MS77	B.1.1.7-2	Δ69–70, Δ144, N501Y	B.1.1.7	Alpha
MS64	B.1.1.7-1	Δ69–70, Δ144, N501Y	B.1.1.7	Alpha
MS59	B.1.1.7-1	Δ69–70, Δ144, N501Y	B.1.1.7	Alpha
MS55	B.1.1.7-2	Δ69–70, Δ144, N501Y	B.1.1.7	Alpha
MS40	B.1.1.7-1	Δ69–70, Δ144, N501Y	B.1.1.7	Alpha
MS33	B.1.1.7-2	Δ69–70, Δ144, N501Y	B.1.1.7	Alpha
MS66	B.1.1.7-1	Δ69–70, Δ144, N5017	B.1.1.7	Alpha
115	B.1.1.7.-2	Δ69–70, Δ144, N501Y	B.1.1.7	Alpha
149	B.1.1.7-1	Δ69–70, Δ144, N501Y	B.1.1.7	Alpha
183	B.1.1.7-2	Δ69–70, Δ144, N501Y	B.1.1.7	Alpha
184	B.1.1.7-1	Δ69–70, Δ144, N501Y	B.1.1.7	Alpha
188	B.1.1.7-2	Δ69–70, Δ144, N501Y	B.1.1.7	Alpha
MS19	B.1.1.7-1	Δ69–70, Δ144, N501Y	B.1.1.7	Alpha
198	B.1.1.7.-2	Δ69–70, Δ144, N501Y	B.1.1.7	Alpha
222	B.1.1.7-1	Δ69–70, Δ144, N501Y	B.1.1.7	Alpha
MS31	B.1.1.7-1	Δ69–70, Δ144, N501Y	B.1.1.7	Alpha
36	B.1.1.7.-2	Δ69–70, Δ144, N501Y	B.1.1.7	Alpha
260	B.1.1.7-1	Δ69–70, Δ144, N501Y	B.1.1.7	Alpha
261 (A54)	B.1.1.7-2	Δ69–70, Δ144, N501Y	B.1.1.7	Alpha
MS27	B.1.1.7-1	Δ69–70, Δ144, N501Y	B.1.1.7	Alpha
236	B.1.1.7-1	Δ69–70, Δ144, N501Y	B.1.1.7	Alpha
MS4	B.1.1.7-1	Δ69–70, Δ144, N501Y	B.1.1.7	Alpha
MS41	B.1.1.7-2	Δ69–70, Δ144, N501Y	B.1.1.7	Alpha
MS43	B.1.1.7-1	Δ69–70, Δ144, N501Y	B.1.1.7	Alpha
44	B.1.1.7-2	Δ69–70, Δ144, N501Y	B.1.1.7	Alpha
24	B.1.1.7-2	Δ69–70, Δ144, N501Y	B.1.1.7	Alpha
89	B.1.1.7-1	Δ69–70, Δ144, N501Y	B.1.1.7	Alpha
91	B.1.1.7-2	Δ69–70, Δ144, N501Y	B.1.1.7	Alpha
MS78	B.1.1.7-1	Δ69–70, Δ144, N501Y	B.1.1.7	Alpha
MS80	B.1.1.7-1	Δ69–70, Δ144, N501Y	B.1.1.7	Alpha
13	B.1.1.7.-1	Δ69–70, Δ144, N501Y	B.1.1.7	Alpha
MS76	B.1.1.7-1	Δ69–70, Δ144, N501Y	B.1.1.7	Alpha
MS62	B.1.1.7-2	Δ69–70, Δ144, N501Y	B.1.1.7	Alpha
MS47	B.1.1.7-2	Δ69–70, Δ144, N501Y	B.1.1.7	Alpha
MS34	B.1.1.7-1	Δ69–70, Δ144, N501Y	B.1.1.7	Alpha
MS24	B.1.1.7-1	Δ69–70, Δ144, N501Y	B.1.1.7	Alpha
MS20	B.1.1.7-2	Δ69–70, Δ144, N501Y	B.1.1.7	Alpha
MS11	B.1.1.7-2	Δ69–70, Δ144, N501Y	B.1.1.7	Alpha
51	B.1.1.7-2	Δ69–70, Δ144, N501Y	B.1.1.7	Alpha
45	B.1.1.7-1	Δ69–70, Δ144, N501Y	B.1.1.7	Alpha
7	B.1.1.7-1	Δ69–70, Δ144, N501Y	B.1.1.7	Alpha
MS10	B.1.1.7-1	Δ69–70, Δ144, N501Y	B.1.1.7	Alpha
114	B.1.1.7-1	Δ69–70, Δ144, N501Y	B.1.1.7	Alpha
163	B.1.1.7-1	Δ69–70, Δ144, N501Y	B.1.1.7	Alpha
227 (A20)	B.1.1.7.-2	Δ69–70, Δ144, N501Y	B.1.1.7	Alpha
MS23	B.1.1.7.-1	Δ69–70, Δ144, N501Y	B.1.1.7	Alpha
171	B.1.1.7-2	Δ69–70, Δ144, N501Y	B.1.1.7	Alpha
173	B.1.1.7-1	Δ69–70, Δ144, N501Y	B.1.1.7	Alpha
150	B.1.1.7-1	Δ69–70, Δ144, N501Y	B.1.1.7	Alpha
190	B.1.1.7-2	Δ69–70, Δ144, N501Y	B.1.1.7	Alpha
14	B.1.1.7-2	Δ69–70, Δ144, N501Y	B.1.1.7	Alpha
199	B.1.1.7-1	Δ69–70, Δ144, N501Y	B.1.1.7	Alpha
MS2	B.1.1.7-1	Δ69–70, Δ144, N501Y	B.1.1.7	Alpha
209	B.1.1.7-1	Δ69–70, Δ144, N501Y	B.1.1.7	Alpha
69	B.1.1.7-2	Δ69–70, Δ144, N501Y	B.1.1.7	Alpha
MS7	B.1.1.7.-1	Δ69–70, Δ144, N501Y	B.1.1.7	Alpha
MS30	B.1.1.7-2	Δ69–70, Δ144, N501Y	B.1.1.7	Alpha
211	B.1.1.7-2	Δ69–70, Δ144, N501Y	B.1.1.7	Alpha
213	B.1.1.7-1	Δ69–70, Δ144, N501Y	B.1.1.7	Alpha
214	B.1.1.7-2	Δ69–70, Δ144, N501Y	B.1.1.7	Alpha
250	B.1.1.7.-2	Δ69–70, Δ144, N501Y	B.1.1.7	Alpha
257	B.1.1.7-1	Δ69–70, Δ144, N501Y	B.1.1.7	Alpha
223 (A16)	B.1.1.7-2	Δ69–70, Δ144, N501Y	B.1.1.7	Alpha
25	B.1.1.7-1	Δ69–70, Δ144, N501Y	B.1.1.7	Alpha
49-2	B.1.1.7-1	Δ69–70, Δ144, N501Y	B.1.1.7	Alpha
46-2	B.1.1.7-1	Δ69–70, Δ144, N501Y	B.1.1.7	Alpha
16-1	B.1.1.7-2	Δ69–70, Δ144, N501Y	B.1.1.7	Alpha
66	B.1.1.7-1	Δ69–70, Δ144, N501Y	B.1.1.7	Alpha
49-1	B.1.1.7-1	Δ69–70, Δ144, N501Y	B.1.1.7	Alpha
95	B.1.1.7-1	Δ69–70, Δ144, N501Y	B.1.1.7	Alpha
260 (A53)	B.1.1.7-2	Δ69–70, Δ144, N501Y	B.1.1.7	Alpha
72	B.1.1.7-2	Δ69–70, Δ144, N501Y	B.1.1.7	Alpha
82	B.1.1.7-1	Δ69–70, Δ144, N501Y	B.1.1.7	Alpha
92	B.1.1.7-1	Δ69–70, Δ144, N501Y	B.1.1.7	Alpha
MS45	B.1.1.7-2	Δ69–70, Δ144, N501Y	B.1.1.7	Alpha
48	B.1.1.7-1	Δ69–70, Δ144, N501Y	B.1.1.7	Alpha
MS5	B.1.1.7-2	Δ69–70, Δ144, N501Y	B.1.1.7	Alpha
50	B.1.1.7-1	Δ69–70, Δ144, N501Y	B.1.1.7	Alpha
52	B.1.1.7-1	Δ69–70, Δ144, N501Y	B.1.1.7	Alpha
223	B.1.1.7.-2	Δ69–70, S98F, Δ144, N501Y	B.1.1.7	Alpha
248	B.1.1.7.-2	Δ69–70, S98F, Δ144, N501Y	B.1.1.7	Alpha
225	B.1.1.7.-2	Δ69–70, Δ144, G181V, N501Y	B.1.1.7	Alpha
225 (A18)	B.1.1.7.-1	Δ69–70, Δ144, G181V, N501Y	B.1.1.7	Alpha
130	B.1.1.7.-2	Δ69–70, Δ144, S155R, N501Y	B.1.1.7	Alpha
231	B.1.1.7.-2	S98F, Δ144, N501Y	B.1.1.7	Alpha
166	B.1.1.7-2	Δ144, N501Y	B.1.1.7	Alpha
105	B.1.1.7-2	Δ144, N501Y	B.1.1.7	Alpha
254	B.1.1.7-2	Δ144, N501Y	B.1.1.7	Alpha
249	B.1.1.7-2	Δ144, N501Y	B.1.1.7	Alpha
88	B.1.1.7-2	Δ144, N501Y	B.1.1.7	Alpha
221	B.1.1.7.-2	Δ69–70, N501Y	B.1.1.7	Alpha
187	B.1.1.7-2	N501Y	B.1.1.7	Alpha
132	B.1.1.7.-2	N501Y	B.1.1.7	Alpha
195	B.1.1.7-2	N501Y	B.1.1.7	Alpha
	Beta (B.1.351)			
215	B.1.351-1-2	D80A, D215G, Δ241–243, K417N, E484K, N501Y	B.1.351	Beta
218	B.1.351-1-2	D80A, D215G, 241–243, K417N, E484K, N501Y	B.1.351	Beta
63	B.1.351-1-2	D80A, D215G, Δ241–243, K417N, E484K, N501Y	B.1.351	Beta
46-1	B.1.351-1-2	D80A, D215G, Δ241–243, K417N, E484K, N501Y	B.1.351	Beta
MS68	B.1.351-1-2	D80A, D215G, Δ241–243, K417N, E484K, N501Y	B.1.351	Beta
	Eta (B.1.525)			
MS37	B.1.525 1-2	A67V, Δ69–70, Δ144, E484K	B.1.525	Eta
	WT			
22	WT	No mutation	WT	WT
80	WT	No mutation	WT	WT
41	WT	No mutation	WT	WT
23	WT	No mutation	WT	WT
10	WT	No mutation	WT	WT
125	WT	No mutation	WT	WT
127	WT	No mutation	WT	WT
128	WT	No mutation	WT	WT
160	WT	No mutation	WT	WT
164	WT	No mutation	WT	WT
17	WT	No mutation	WT	WT
18	WT	No mutation	WT	WT
6	WT	No mutation	WT	WT
37	WT	No mutation	WT	WT
30	WT	No mutation	WT	WT
5	WT	No mutation	WT	WT
M16	WT	No mutation	WT	WT
1	WT	No mutation	WT	WT
21	WT	No mutation	WT	WT
2	WT	No mutation	WT	WT
Dissimilarity (n = 22, 15%)				
94	B.1.1.7.-1	Δ69–70, Δ142, Y144V, N501Y	Missense mutation	Missense mutation
42	B.1.1.7.-1	Δ69–70, Δ142, Y144V, N501Y	Missense mutation	Missense mutation
MS44	B.1.1.7.-1	Δ69–70, Δ144, V289L, N501Y	Missense mutation	Missense mutation
MS49	B.1.1.7.-2	Δ69–70, L141F, Δ144, N501Y	Missense mutation	Missense mutation
99	B.1.1.7.-2	Δ69–70, Δ144, S155R, F374S, N501Y	Missense mutation	Missense mutation
MS65	B.1.1.7.-2	A67V, Δ69–70, Δ144, N5017	B.1.525	Eta
39	B.1.1.7.-2	L452R, N501Y	B.1.617	Delta
27	B.1.1.7.-2	No mutation	No mutation	No mutation
MS51	WT	V213V_RTD, Q414K, N450K	Missense mutation	Missense mutation
78	WT	M153T, Y508H	Missense mutation	Missense mutation
129	WT	M153T, Y508H	Missense mutation	Missense mutation
16-2	WT	Δ144, V320F	Missense mutation	Missense mutation
43	WT	I101T	Missense mutation	Missense mutation
15	WT	M153T	Missense mutation	Missense mutation
28	WT	Δ144	Missense mutation	Missense mutation
29	WT	Δ144	Missense mutation	Missense mutation
26	WT	Δ144	Missense mutation	Missense mutation
19	WT	Δ144	Missense mutation	Missense mutation
4	WT	T478K	B.1.617.2	Delta
178	WT	A222V	B.617.2	Delta
70	WT	T478K	B.1.617.2	Delta
M24	WT	F157S, A520S	B.1.526.1	Lota

CoV-RDB: Coronavirus Antiviral and Resistance Database; WHO: The World Health Organization.

Using phylogenetic analysis, three lineages, including B.1.1.7-1/2 (alpha; n = 103, 72%), B.1.351 (beta; n = 5, 3%) and B.1.525 (eta; n = 1, 1%) were identified among 109 SARS-CoV-2 variations. Using the CoV-RDB, five different lineages involving B.1.1.7 (alpha; n = 95, 66%), B.1.351 (beta; n = 5, 4%), B.1.617 (delta; n = 4, 3%), B.1.525 (eta; n = 2, 1.4%), B.1.526-1 (lota; n = 1, 0.6%) and missense mutations (n = 15, 10%) were reported. The most frequent *S-*region variation pattern was Δ69–70, Δ144, N501Y. A (D80A, D215G, Δ241–243, K417N, E484K, N501Y) mutation pattern was the only variation noted in B.1.351. Moreover, the variations (T478K; n = 2, 1.6%), (L452R, N501Y; n = 1, 0.8%) and (A222V; n = 1, 0.8%) were also identified less frequently by CoV-RDB analysis. We also determined an (A67V, Δ69–70, Δ144, E484K) mutation pattern in one strain. Mutation patterns/amino acid substitutions (Δ69–70, Δ142, Y144V, N501Y), (Δ69–70, Δ144, V289L, N501Y), (Δ69–70, L141F, Δ144, N501Y), (Δ69–70, Δ144, S155R, F374S, N501Y), (V213V_RTD, Q414K, N450K), (M153T, Y508H), (Δ144, V320F), (I101T), (M153T) and (Δ144) were considered missense mutations, as they involve different amino acid changes for which the impact has not been well identified.

The distribution of SARS-CoV-2 variations as lineages and amino acid mutations identified by phylogenetic analysis and using the CoV-RDB is given in Table 1. When the variations obtained by CoV-RDB were compared with the variations obtained by phylogenetic analysis, a similarity rate of 121 (85%) was observed in the genome analysis of the two variant detection methods. The highest similarity was observed in the identification of B.1.1.351 (100%), followed by B.1.1.7 (92%), by the two methods. Similarity rates of SARS-CoV-2 variations by phylogenetic analysis and CoV-RDB are given in Table 2. Consequently, B.1.1.7 (alpha) was the most frequent SARS-CoC-2 variation in Turkey in April 2021.

**Table 2.** The rate of identified mutations by phylogenetic analysis and Coronavirus Antiviral and Resistance Database analysis.

Variant	Phylogenetic analysis, n (%)	CoV-RDB, n (%)	Similarity rate, (%)
VOC			
B.1.1.7 (alpha)	103 (72%)	95 (66%)	92%
B.1.350 (beta)	5 (3%)	5 (4%)	100%
P.1 (gamma)	None	None	–
B.617 (delta)	None	4 (3%)	No similarity
VOI			
Epsilon	None	None	–
Zeta	None	None	–
Eta	1 (1%)	2 (1.4%)	50%
Theta	None	None	–
Lota	None	1 (0.6%)	No similarity
Kappa	None	None	–
Lambda	None	None	–
WT	34 (24%)	21 (15%)	62%
Missense mutation	None	15 (10%)	No similarity
Total	143	143	–

CoV-RDB: Coronavirus Antiviral and Resistance Database; VOC: Variant of concern; VOI: Variant of interest; WT: Wild type.

## Discussion

Continuous description of the genomic characterization of SARS-CoV-2 followed by variant analysis with powerful online tools is crucial, as it provides important information on changes in COVID-19 epidemiology, clinical disease outcomes and efficiency of diagnostics, vaccines and therapeutics, due to viral genome diversity [23]. In the current study, we sequenced the *spike* gene of SARS-CoV-2 strains of COVID-19-infected cases in Turkey in April 2021, as the *S* gene is key for SARS-CoV-2 surveillance to identify nucleotide variations [15,24–26]. In SARS-CoV-2 *spike* genomes, we reported 76% and 85% nucleotide variations by phylogenetic analysis and CoV-RDB analysis, respectively.

The genomic findings revealed that although two major VOCs, including B.1.1.7-1/2 (alpha), B.1.351 (beta), and one VOI (B.1.525) were circulating in Turkey in April 2021. B.1.1.7 (501Y.V1) was determined to be dominant in the population during that period of time. B.1.1.7 (501Y.V1) was first designated in the United Kingdom in December 2020, and subsequently spread across the world to countries including the United States, Mexico, Brazil, Argentine, Spain, Germany, South Africa, Saudi Arabia, Pakistan, Bulgaria, Russia, India, China, Australia and so on. [2]. Globally, as of April 27, 2021, 501Y.V1 has been reported in 139 countries, followed by 501Y.V2 (beta) and 501Y.P1 (gamma) in 87 and 54 countries, respectively [27]. As SARS-CoV-2 is in circulation, it keeps evolving. Recently, B.1.617 (delta) has become the predominant variation worldwide. The delta variant was first reported in India in October 2020 [2]. By July 13, 2021, 111 countries had reported cases of the delta variant [28]. After two weeks, it had spread to 132 countries [29] and by August 17, 2021, 148 countries confirmed the presence of the delta variant [30]. Tracking changes in the SARS-CoV-2 *spike* reveals that SARS-CoV-2 variations should be monitored continuously by genome sequence analysis in Turkey and in other countries.

During the pandemic, it is important to identify variants as quickly as possible. In this study, we evaluated the sequenced data for SARS-CoV-2 variations by two different variant detection methods to better understand the diagnostic power of tools commonly used in variant analysis. As there are no data in the literature that reflect this comparison, we evaluated the detection performance of a virtual phenotyping method with the gold standard method, phylogenetic analysis. The findings showed that the two sequence analysis methods were 85% compatible. Interestingly, we reported the highest similarity in the identification of B.1.1.351 (100%), followed by B.1.1.7 (92%) by two methods. The similarity of the results suggests that the CoV-RDB, which provides more rapid sequence exploring, may also be an alternative appropriate approach in determining SARS-CoV-2 mutations.

Although *spike* sequencing and analysis are used as the gold standard for accurate genomic surveillance, SARS-CoV-2 PCR variant screening kits were performed before NGS to distinguish particular SARS-CoV-2 variants circulating in Turkey among all SARS-CoV-2 PCR-positive cases. The current findings clarified that 24% and 15% of the strains were identified as wildtype by phylogenetic analysis and CoV-RDB, respectively, although these strains were determined as SARS-CoV-2 variants by multiplex PCR kits. Durner *et al.* demonstrated the feasibility of Y501 variant-specific PCR for fast and reliable detection of UK SARS-CoV-2 variants in routine diagnosis, and their suspected variant was confirmed by the reference laboratory [31]. Similarly, Zhao *et al.* provided both the specificity and the sensitivity of the SARS-CoV-2 variants based on multiplex PCR-matrix-assisted laser desorption ionization-time of flight mass spectrometry (MALDI-TOF-MS) at 100% [32]. In another study, the positive and negative predictive values were 100% for RT-qPCR assay for screening the *spike* N501Y mutation [33]. According to the current findings, variant screening PCR kits could be good alternative choices to detect variant strains for NGS analysis, which enables saving time and cost, especially for developing countries.

To point out the limitations of this study, our genomic analysis identifies variations of cases infected with COVID-19 only in the provinces of Istanbul, Kocaeli and Ankara in April 2021. To reveal the genomic variations of SARS-CoV-2 in the whole of Turkey, more cases from many different cities should be included and these cases should be investigated periodically to provide updated surveillance.

## Conclusion

In the COVID-19 pandemic, variant emergence is possible and may be rapid. Therefore, SARS-CoV-2 strains should be constantly monitored. Phylogenetic analysis and Stanford CoV-RDB analysis methods seem useful for this surveillance.

**Summary points**

- Genomic characterization of SARS-CoV-2 allows the description of important information on phenotypic characteristics, including disease transmission, disease severity, diagnostic escape and immune escape due to emerging new coronavirus variants.
- Next-generation sequencing is widely used for genomic characterization of SARS-CoV-2, followed by variant analysis with phylogenetic analysis.
- With the rapid design of new virtual phenotyping technologies, identification of SARS-CoV-2 mutations can also be achieved in a short time and at low cost.
- B.1.1.7 (alpha) was the most frequent SARS-CoV-2 variation in Turkey in April 2021.
- The Coronavirus Antiviral and Resistance Database (CoV-RDB) by Stanford University that is freely accessible at https://covdb.stanford.edu/, has been designed to promote comparisons between different candidate compounds against COVID-19, as well as rapid large-scale identification of SARS-CoV-2 mutations, since August 2020.
- The current findings showed that both sequence analysis methods were 85% compatible.
- Phylogenetic analysis and Stanford CoV-RDB analysis methods seem useful for tracking SARS-CoV-2 strains.
